# Caveolae-mediated albumin transcytosis is enhanced in dengue-infected human endothelial cells: A model of vascular leakage in dengue hemorrhagic fever

**DOI:** 10.1038/srep31855

**Published:** 2016-08-22

**Authors:** Chanettee Chanthick, Rattiyaporn Kanlaya, Rattanaporn Kiatbumrung, Sa-nga Pattanakitsakul, Visith Thongboonkerd

**Affiliations:** 1 Molecular Medicine Unit, Office for Research and Development, Faculty of Medicine Siriraj Hospital, Mahidol University, Bangkok, Thailand; 2 Graduate Program in Immunology, Department of Immunology, Faculty of Medicine Siriraj Hospital, Mahidol University, Bangkok, Thailand; 3 Medical Proteomics Unit, Office for Research and Development, Faculty of Medicine Siriraj Hospital, Mahidol University, Bangkok, Thailand; 4 Center for Research in Complex Systems Science (CRCSS), Mahidol University, Bangkok, Thailand

## Abstract

Vascular leakage is a life-threatening complication of dengue virus (DENV) infection. Previously, association between “paracellular” endothelial hyperpermeability and plasma leakage had been extensively investigated. However, whether “transcellular” endothelial leakage is involved in dengue hemorrhagic fever (DHF) and dengue shock syndrome (DSS) remained unknown. We thus investigated effects of DENV (serotype 2) infection on transcellular transport of albumin, the main oncotic plasma protein, through human endothelial cell monolayer by Western blotting, immunofluorescence staining, fluorescence imaging, and fluorometry. The data showed that Alexa488-conjugated bovine serum albumin (Alexa488-BSA) was detectable inside DENV2-infected cells and its level was progressively increased during 48-h post-infection. While paracellular transport could be excluded using FITC-conjugated dextran, Alexa488-BSA was progressively increased and decreased in lower and upper chambers of Transwell, respectively. Pretreatment with nystatin, an inhibitor of caveolae-dependent endocytic pathway, significantly decreased albumin internalization into the DENV2-infected cells, whereas inhibitors of other endocytic pathways showed no significant effects. Co-localization of the internalized Alexa488-BSA and caveolin-1 was also observed. Our findings indicate that DENV infection enhances caveolae-mediated albumin transcytosis through human endothelial cells that may ultimately induce plasma leakage from intravascular compartment. Further elucidation of this model *in vivo* may lead to effective prevention and better therapeutic outcome of DHF/DSS.

Dengue virus (DENV), a member of genus *Flavivirus* in the family *Flaviviridae*, causes a mosquito-borne disease that is one of leading public health problems of the 21^st^ century^1^. Approximately 2.5 billion people in more than 100 tropical and subtropical countries are at risk of DENV infection^1^. The most severe form of this infectious disease is associated with various degrees of plasma leakage at the end of febrile phase, namely dengue hemorrhagic fever (DHF), which can further develop to the fatal dengue shock syndrome (DSS)^2^,^3^. Because initial symptoms in febrile phase of DHF/DSS are difficult to be distinguished from the mild form, dengue fever (DF), together with the unclear disease mechanisms, occurrence of the life-threatening plasma leakage is hard to predict and becomes the obstacle in management of DHF/DSS^4^,^5^.

Vascular wall, as the target or affected tissue for plasma leakage, is composed of endothelial cell monolayer that acts as a semi-permeable barrier. Endothelial cell has an ability to regulate its paracellular (through intercellular junctions) and transcellular (through endothelial cell) transports of fluid, solutes, and proteins^6^,^7^. The association between paracellular endothelial hyperpermeability and plasma leakage has been extensively investigated in several diseases, including DENV infection^8^,^9^,^10^,^11^. Previous studies have postulated that immunological host response can enhance the disruption of endothelial intercellular junctions, resulting in paracellular leakage of plasma from intravascular space to serous cavities^8^,^9^,^10^,^11^. However, little is known about involvement of transcellular hyperpermeability in the disease pathogenesis.

Transcellular albumin transport or albumin transcytosis is a common feature of endothelial cell that also serves as a key mechanism to regulate basal vascular permeability (BVP) under normal condition^12^,^13^,^14^. Albumin, the main oncotic molecule in plasma, is a negatively charged protein that can attract cations, e.g. sodium (Na^+^), and water to follow and move across vascular endothelium. To maintain intravascular plasma fluid volume and to supply essential nutrients, only a limited amount of albumin is permitted to extravasate via the transcellular mechanism^12^,^13^,^14^. The extravasation rate is kept in balance with fluid reabsorption and lymphatic drainage rate to prevent extravascular fluid accumulation^14^. The transcellular endothelial hyperpermeability has been proposed to be associated with pathogenesis of some diseases^15^. For example, in acute lung injury, the increase of caveolae-mediated transendothelial albumin transport is initiated by adherence of fMLP-activated neutrophils to vascular wall, resulting to fluid imbalance and excessive extravascular fluid accumulation^16^. However, whether transcellular endothelial leakage exists and is involved in DHF and DSS remained unknown. The present study thus aimed to address effects of DENV infection on albumin transcytosis in human endothelial (EA.hy926) cells that could be linked to the pathogenic mechanisms of plasma leakage in DHF/DSS.

## Results

Because albumin transcytosis is a transport of albumin cargo across endothelial cell from luminal (intravascular) to abluminal (extravascular) space, we used the Transwell system to examine this mechanistic process. Crucial criteria for the *in vitro* transcytosis model^17^, including intracellular accumulation of cargo, integrity of cell monolayer, cargo uptake from one side (upper chamber) (representing luminal/intravascular space) of the Transwell and its appearance in the opposite side (lower chamber) (representing abluminal/extravascular space), were altogether investigated.

### DENV2 infection enhanced internalization of albumin into EA.hy926 human endothelial cells

In this study, our concern was that the degree of DENV2 infection, represented by multiplicity of infection (MOI) used for DENV2 challenge and post-infection incubation period, should not induce obvious cytotoxicity that increased cell death (as we wished to address specific host cellular response, not the effects of cell death that were rather non-specific). We thus carefully screened for such optimal condition that could provide specific host cellular response while cell death remained unchanged when compared to the mock-control. The data showed that the MOI of 2.0 and post-infection incubation of up to 48 h was the optimal condition for our present study (see Supplementary Fig. S1). Therefore, the MOI of 2.0 and up to 48 h post-infection incubation period were used throughout the study.

**Figure 1:**
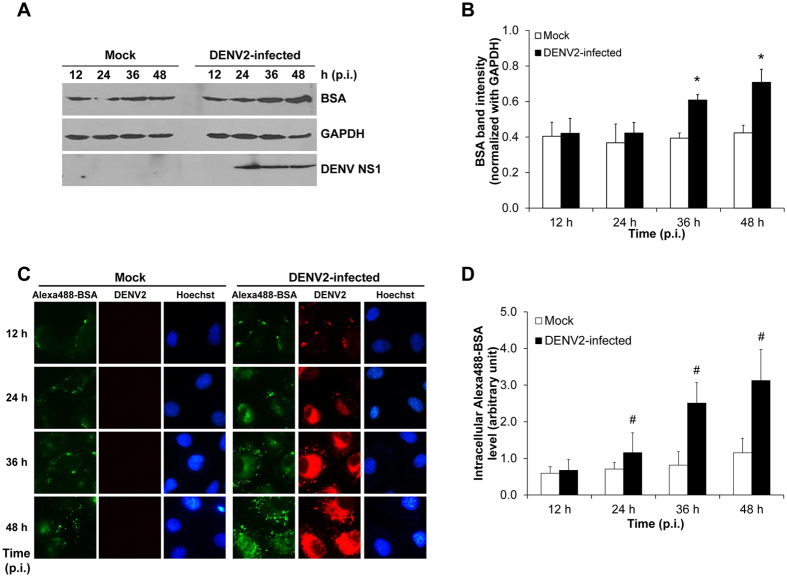
Enhanced internalization of BSA into DENV2-infected human endothelial cells. **(A)** Intracellular BSA in DENV2-infected EA.hy926 cells at various post-infection time-points was determined by Western blot analysis. GAPDH served as the loading control while non-structural 1 (NS1) was a marker for DENV infection. **(B)** Quantitative analysis of BSA band intensity **(C)** Detection of BSA conjugated with Alexa Fluor 488 (Alexa488-BSA) (shown in green) by a laser scanning confocal microscope (LSM 510 Meta, Carl Zeiss). Hoechst staining was performed to demonstrate nuclei. DENV stained in red served as the marker for successful DENV2 infection. Original magnification was 630X for all panels. **(D)** Fluorescence intensity of the internalized Alexa488-BSA was quantitated using ImageJ software. (n = 3 independent experiments for both Western blot analysis and fluorescence study; *p < 0.05 vs. mock control; ^#^*p* < 0.01 vs. mock control).

To determine the enhanced intracellular accumulation of albumin in human endothelial cells during DENV2 infection, Western blot analysis was performed. BSA, which was originated from bovine, was supposed to be transported increasingly inward EA.hy926 human endothelial cells upon DENV infection. The data revealed that albumin internalization was observed in both mock and DENV2-infected cells, and was significantly increased in DENV2-infected cells at 36 and 48 h post-infection (Fig. 1A,B). Using more sensitive techniques, fluorescence tag and laser scanning confocal microscopy, the intracellular accumulation of Alexa488-BSA was progressively increased in DENV2-infected cells at 24, 36 and 48 h post-infection, whereas that in the mock-control cells tended to slightly increase at 48 h post-infection but did not reach statistically significant threshold (Fig. 1C,D). These findings indicated that DENV infection significantly increased albumin internalization into human endothelial cells.

### Survival and morphology of EA.hy926 human endothelial cells were not affected by DENV2 infection at MOI of 2.0

**Figure 2:**
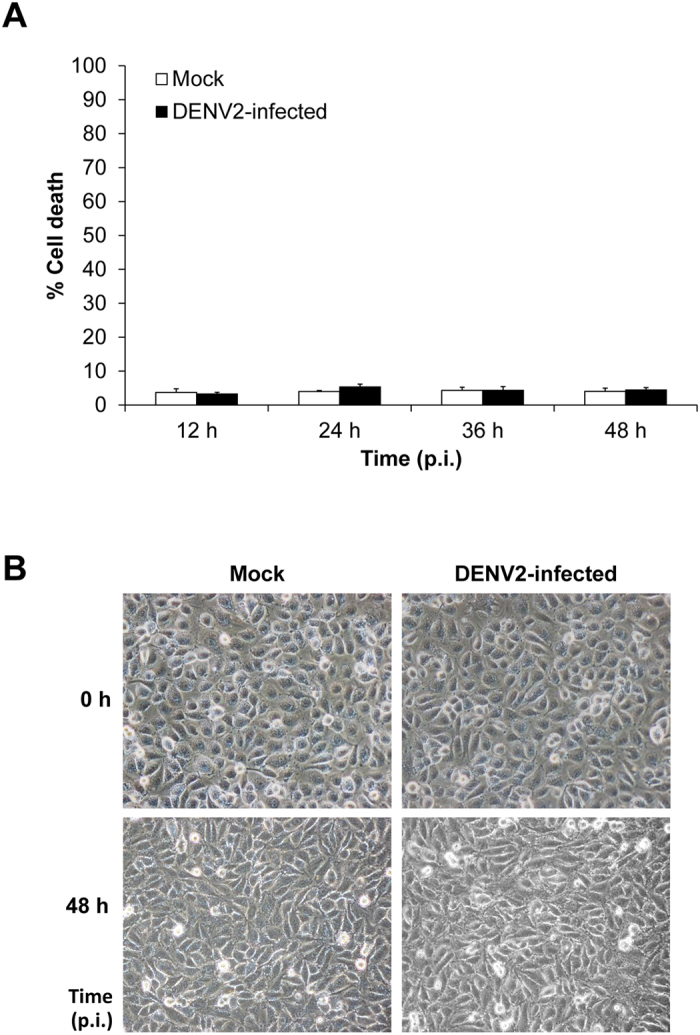
EA.hy926 cell monolayer was intact after infection with DENV2. **(A)** Cell death was quantitated by flow cytometry using Annexin V/propidium iodide co-staining. Percentage of cell death was calculated using the formula: % cell death = [(no. of total cell death (apoptosis + necrosis)/no. of total cells) × 100%] (n = 3 independent experiments). **(B)** At 0 to 48 h post-infection, the morphology of mock-control and DENV2-infected cells was analyzed using an inverted light microscope. (Original magnification was 400X).

Because dead cells might affect integrity of the cellular monolayer by losing their contact with the adjacent cells^18^, morphology and cell death of EA.hy926 monolayer in the Transwell insert were examined. By flow cytometric analysis, the data revealed comparable percentage of cell death between mock-control and DENV2-infected cells at all time-points (12, 24, 36 and 48 h post-infection) (Fig. 2A). Under an inverted light microscope, the morphology of mock-control and DENV2-infected cells looked similar and had no obvious differences at all time-points (Fig. 2B).

### Increased albumin transcytosis (permeated albumin) through DENV2-infected endothelial cells

**Figure 3:**
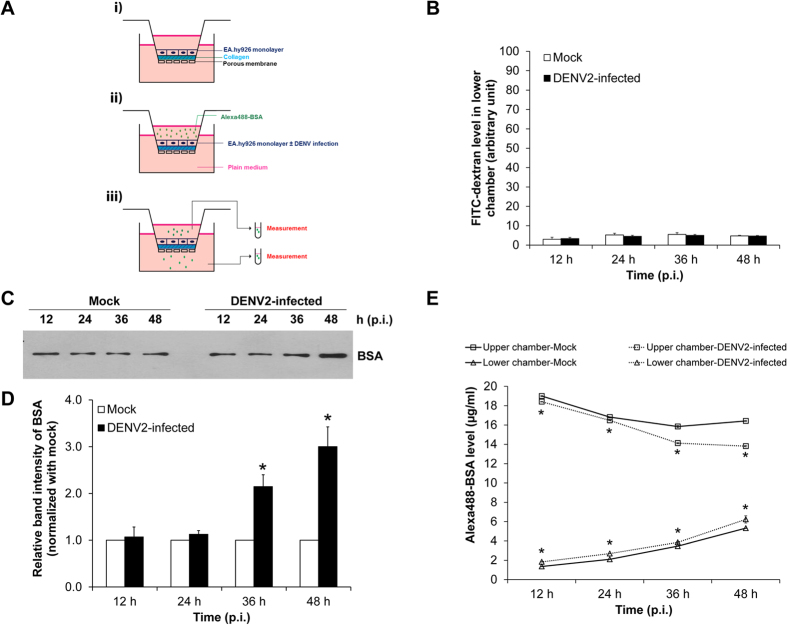
Integrity of EA.hy926 cell monolayer was intact, whereas albumin transcytosis was increased in DENV2-infected cells. **(A)** A schematic diagram of the Transwell-based transcytosis assay. i) EA.hy926 cells form a monolayer on a collagen-coated membrane filter. ii) Alexa488-BSA was added into the insert well (upper chamber) of the Transwell chamber. iii) Alexa488-BSA remained in the upper chamber and that passed through endothelial monolayer into the lower chamber were measured. **(B)** The integrity of endothelial cell monolayer was evaluated by *in vitro* vascular permeability assay. Level of the permeated dextran conjugated with FITC (FITC-dextran) in the lower chamber, which reflects degree of permeability of the Transwell, was measured by SynergyHT microplate reader **(C,D)** BSA passed through the cells into the lower chamber was analyzed by Western blotting using rabbit polyclonal anti-BSA as the primary antibody. **(E)** Alexa488-BSA remained in the upper chamber and that passed through endothelial monolayer into the lower chamber were measured by fluorometer. (n = 3 independent experiments both Western blot analysis and fluorometry; *p < 0.05 vs. mock-control).

To evaluate albumin transcytosis, EA.hy926 cells were seeded onto collagen-coated membrane filter and Alexa488-BSA was added into upper chamber of the Transwell system (Fig. 3A). The degree of albumin transcytosis was then examined by measuring Alexa488-BSA in upper and lower chambers using fluorometry (Fig. 3A). To ensure that transcellular albumin transport was not mixed up with paracellular pathway, the integrity of endothelial monolayer was evaluated by an *in vitro* vascular permeability assay. FITC-dextran (a macromolecule with a size larger than 3 nm) permeated from upper chamber (through paracellular pathway) into plate well (lower chamber) was measured. The data showed that FITC-dextran level in lower chamber of the DENV2-infected group was comparable to that of the mock-control (Fig. 3B). This data indicated that the integrity of endothelial monolayer was not affected by DENV infection at the MOI of 2.0 and effect from paracellular pathway could be ruled out from the *in vitro* albumin transcytosis assay in our present study. While the integrity and paracellular permeability of EA.hy926 human endothelial monolayer were preserved, permeability of BSA from one side (upper chamber) (representing luminal/intravascular space) of the Transwell to the opposite side (lower chamber) (representing abluminal/extravascular space) was evaluated first by Western blot analysis. The data revealed that DENV2-infected cells had greater BSA level permeated through the cells (transcytosis) at 36 and 48 h post-infection (Fig. 3C,D). In addition, level of permeated or transcytosed Alexa488-BSA was also measured using a more sensitive technique – fluorometry. In DENV2-infected cells, while Alexa488-BSA level in upper chamber was progressively decreased, its level in lower chamber was progressively increased. Comparing to the mock-control, such decrease and increase in upper and lower chambers, respectively, were greater in DENV2-infected cells at all time-points (Fig. 3E). Collectively, our results demonstrated that DENV infection increased transcellular albumin transport in human endothelial cells.

### Albumin internalization in DENV2-infected endothelial cells was mediated by caveolae-dependent endocytic pathway

**Figure 4:**
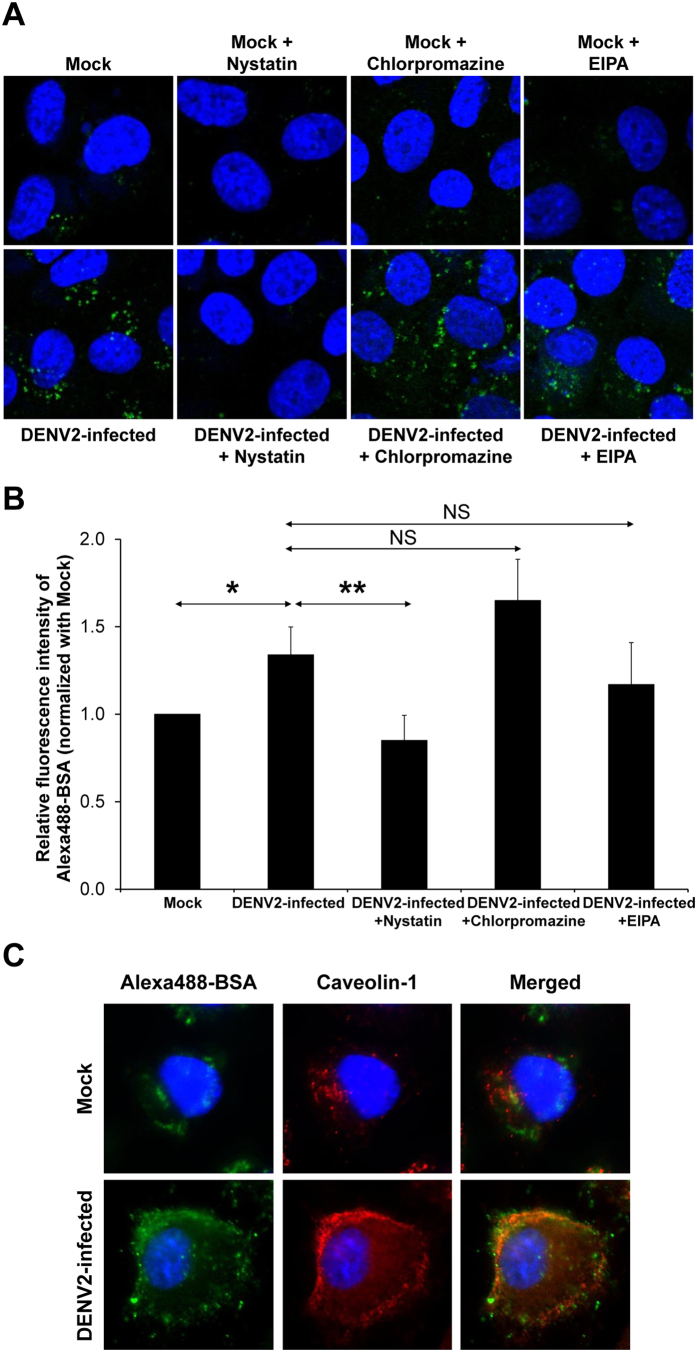
Effect of inhibitors of individual endocytic pathways on albumin transcytosis in DENV2-infected EA.hy926 cells. At 47 h post-infection, the inhibitor (nystatin, chlorpromazine or EIPA) was pretreated with the cells for 10 min prior to incubation with Alexa488-BSA for 50 min. **(A)** The internalized Alexa488-BSA (shown in green) was imaged by a laser scanning confocal microscope (LSM 510 Meta, Carl Zeiss) (original magnification was 630X). **(B)** The intensity of the internalized Alexa488-BSA was quantitatively by using Axiovision software (n = 3 independent experiments; *p < 0.0005; **p < 0.0001; NS = not significant). **(C)** Immunofluorescence co-staining of Alexa488-BSA (in green) and caveolin-1 (in red). The images were captured by a laser scanning confocal microscope (LSM 510 Meta, Carl Zeiss) (Hoechst staining was performed to demonstrate nuclei) (original magnification was 630X).

Endocytosis has been proposed to be the responsible mechanism of albumin transcytosis. To define the endocytic or cellular entry pathway induced by DENV infection, the well known endocytic pathways, including caveolae-dependent endocytosis, clathrin-coated vesicle-mediated endocytosis and macropinocytosis, were examined using corresponding pharmacological inhibitors (Fig. 4A). Quantitative analysis of intracellular level of Alexa488-BSA after pretreatment of DENV2-infected endothelial cells with nystatin, the inhibitor of caveolae-mediated endocytosis, showed significant decrease of albumin internalization by approximately 37% of that in the DENV2-infected cells without nystatin pretreatment (Fig. 4B). The cells pretreated with chlorpromazine and EIPA (the inhibitors of clathrin-coated vesicle-mediated endocytosis and macropinocytosis, respectively) tended to have increased and decreased albumin internalization, respectively. However, those tendencies did not reach statistically significant threshold (Fig. 4B). To confirm the inhibitory effect of nystatin on albumin internalization, co-localization of the internalized Alexa488-BSA and caveolin-1 (a protein marker for caveolae) was evaluated by immunofluorescence study. While such co-localization was not obvious in the cock-control cells, it was striking in the DENV2-infected cells (Fig. 4C). Moreover, the data also revealed increased expression level of caveloin-1 in the DENV2-infected cells (Fig. 4C). These datasets strongly indicated that albumin internalization in DENV2-infected endothelial cells was mediated by caveolae-dependent endocytic pathway.

## Discussion

After infection, DF and DHF patients generally undergo a febrile phase presenting with fever, hepatomegaly, thrombocytopenia, and bleeding tendency. Whereas DF patients can spontaneously recover from this febrile phase, DHF patients develop various degrees of plasma leakage mostly into serous cavities^2^. Plasma leakage progresses from febrile phase and becomes most severe in defervescence phase^19^. This has been evidenced by chest radiography, which shows detectable pleural effusion on the first day of fever and progressive accumulation during the first week^20^. The apparent effusion together with hemoconcentration is commonly maximal during the 4^th^–7^th^ days after the fever onset^21^,^22^,^23^. By ^131^I-HSA estimation, hypovolemic shock may accompany the loss of plasma volume up to 20%^19^. Under close monitoring and prompt fluid therapy, hypovolemic shock persists only within a short period (24–48 h) followed by gradual reabsorption of effusion. Moreover, the recovery of leakage is usually spontaneous without sequelae.

Under normal physiologic condition, modest or limited degree of vascular leakage may occur for supplying nutrients to surrounding tissues^7^,^24^,^25^,^26^. This physiologic leakage is called “basal vascular permeability” (BVP) that is characterized by fast paracellular leakage of water and small molecules, together with tightly regulated transcellular passage of albumin and its cargo, caveolae, the plasmalemmal vesicles that can shuttle across endothelial cell from luminal to abluminal side^6^,^12^,^14^,^27^,^28^,^29^,^30^,^31^. However, the transcellular BVP is not generalized for all compositions of the plasma but is, indeed, selective to albumin and albumin-bound substances (e.g., amino acids, fatty acids, hormones, etc.). In human, albumin is the most abundant plasma protein (over a half of all plasma proteins), whereas other major abundant proteins (i.e. globulin and fibrinogen) occupy only 38% and 7%, respectively. Its highly negative charges can attract cations and water to follow and move across vascular endothelium^32^. These properties highlight albumin to be the main oncotic agent, providing 75% (21.8 mmHg) of total plasma oncotic pressure (28 mmHg)^32^. As such, albumin and its cargoes, which are released by BVP into extravascular compartment, can cause a rapid flux of water and small solutes across the normal vessel^6^,^12^,^14^,^27^,^28^,^29^,^30^,^31^. The outcome is filtration of plasma fluid consisting mainly of water and small-size solutes with tiny amounts of the leaked macromolecules and proteins^24^,^25^. However, the extravasation rate of albumin and plasma filtrate is usually in balance with fluid reabsorption and lymphatic drainage rate to prevent extravascular fluid accumulation^14^.

In DHF and DSS, the association between paracellular endothelial hyperpermeability and plasma leakage had been extensively investigated^8^,^9^,^10^,^11^. However, whether transcellular endothelial leakage exists and is involved in DHF and DSS remained unknown. In the present study, we used a Transwell system to demonstrate the increase of albumin transcytosis in DENV2-infected endothelial cells in comparison with the mock-control cells. Intracellular accumulation of BSA, integrity of endothelial monolayer, and BSA uptake from luminal side (upper chamber) and its appearance on abluminal side (lower chamber) were investigated following the essential criteria for the *in vitro* transcytotic cell model^17^. For the integrity of endothelial monolayer, three indicators including morphology, percentage of cell death and paracellular permeability of DENV2-infected endothelial monolayer were examined in comparison to the mock-control. While the integrity of DENV2-infected endothelial monolayer was intact (Fig. 2), the significantly increased levels of internalized BSA inside the cells (Fig. 1) and passed-through BSA in the lower chamber (Fig. 3) of the DENV2-infected endothelial monolayer indicated that DENV infection had a significant effect on endothelial cell function by enhancing transcellular transport (transcytosis) of albumin.

We next examined whether the enhanced albumin transcytosis was mediated through any endocytic pathway(s). Beside the mostly active caveolae-mediated endocytosis, two other endocytic pathways that have been well defined in endothelial cells, including clathrin-coated pit and macropinocytosis^33^, were also investigated. By the pharmacological approach, albumin internalization, the early step of albumin transcytosis, in DENV2-infected endothelial monolayer was not suppressed by the inhibitors of clathrin-coated pit and macropinocytosis. In contrast, it was significantly inhibited by nystatin (Fig. 4), the cholesterol-sequestering agent that distorts caveolae structure and function^34^. In addition, immunofluorescence co-staining also showed co-localization of Alexa488-BSA and caveolin-1 in the DENV2-infected cells (Fig. 4C). Hence, the increased albumin transcytosis in DENV2-infected endothelial monolayer is caveolae-dependent, implying that DENV infection disturbs the inherent mechanism of BVP. Although caveolae-dependent pathway could not be entirely ruled out in the normal (uninfected) endothelial cells (Fig. 4C), the data suggested that there should be other endocytic pathways that predominately regulate albumin transcytosis in normal endothelial cells, whereas caveolae-dependent pathway is enhanced by DENV infection. Our *in vitro* data were consistent with previous pathological findings, in which electron microscopy of dermal vessels in 60 DHF patients, most of whom endothelial junctional complexes were intact, revealed the prominent increase of pinocytotic/endocytic vesicles in cytoplasm of endothelial cells^35^.

The proposed mechanisms of the enhanced albumin transcytosis induced by DENV infection are discussed as follows. At basal level, albumin transcytosis is initiated by the binding of albumin to its receptor, a 60-kDa glycoprotein (gp60) on endothelial luminal surface^36^,^37^. After binding, gp60 clustering and its interaction with caveolin-1, the primary structural protein of caveolae, activates Src tyrosine kinase, the key switch that phosphorylates caveolin-1 and the scission protein, dynamin-2, to release caveolae from plasma membrane and induce transcellular transport of receptor-bound and fluid phase albumin^15^,^38^,^39^,^40^. In DENV infection, albumin transcytosis is enhanced via caveolae-mediated endocytic vesicular transport, together with the increase in paracellular permeability, both of which mediate vascular leakage and excessive extravascular fluid accumulation. Our proposed mechanisms for the enhanced transcellular permeability are similar to the effects of other pathological and non-pathological stimuli, including neutrophil-endothelial interaction^16^, oxidant^41^, and isoflurane^42^, which have been reported to also enhance caveolae-mediated transendothelial albumin transport.

It is generally accepted that plasma levels of various cytokines and chemokines, e.g., interleukin (IL)-2, IL-6, IL-8, IL-10, IL-12, IL-18, transforming growth factor-β1, interferon-γ and tumor necrosis factor-α, are significantly increased in DHF/DSS patients (known as cytokine storm phenomenon). These mediators have ability to activate paracellular permeability through the actin-based system regulators (e.g., protein kinase C, myosin light-chain kinase, etc.), resulting to reversible endothelial contraction, dissociation/reassembly of junctional protein complex, and finally a rapid efflux of plasma fluid to extravascular compartment. However, to develop extreme loss of plasma volume, the leakage may take place in several pathways. As seen in acute lung injury model and respiratory distress syndrome with severe pulmonary edema, the activated neutrophils increase paracellular permeability by releasing mediators (oxidants and proteases) that disrupt interendothelial junctions. Moreover, adhesion of neutrophils to endothelial cells also increases transcellular permeability by enhancing caveolae-mediated albumin transcytosis, resulting to interstitial edema^16^. Hence, paracellular leakage and transcellular hyperpermeability play a combined role in the disease pathogenesis.

In summary, we have demonstrated for the first time that DENV infection enhances caveolae-mediated albumin transcytosis in human endothelial cells. In addition to the well known paracellular endothelial hyperpermeability mediated by immune response, DENV infection also affects the BVP by an increase in caveolae-mediated transcellular transport of albumin that can further induce plasma leakage from intravascular compartment to extracellular space, leading to DHF/DSS. Further elucidation of this model *in vivo* may lead to effective prevention and better therapeutic outcome of DHF/DSS.

## Materials and Methods

### Cell culture

Human endothelial cell line (EA.hy926) was grown in a complete DMEM/F-12 medium (Gibco; Grand Island, NY), which was supplemented with 10% heat-inactivated fetal bovine serum (FBS) (Gibco), 100 U/ml penicillin G and 100 μg/ml streptomycin (Sigma; St.Louis, MO). The cells were maintained in a humidified incubator with 5% CO_2_ at 37 °C.

C6/36 (derived from *Aedes albopictus*) and swine fibroblast (PScloneD) cell lines, were grown in complete L-15 medium (Gibco) containing 10% tryptose phosphate broth (TPB) (Sigma), 10% FBS, 100 U/ml penicillin G and 100 μg/ml streptomycin. The cells were maintained in a humidified incubator with 5% CO_2_ at 28 °C and 37 °C, respectively.

### Propagation of virus stock and virus titration

DENV serotype 2 (DENV2) (strain 16681) was propagated in C6/36 cells as described previously^43^,^44^. Briefly, the confluent monolayer of C6/36 was incubated with DENV2 at a multiplicity of infection (MOI) of 0.1 in a maintenance L-15 medium (containing 10% TPB, 1% FBS, 100 U/ml penicillin and 100 μg/ml streptomycin) at 28 °C for 3 h with gently continuous shaking. Supernatant was then removed and replaced with fresh maintenance medium, and further incubated at 28 °C until 50% cytopathic effect (CPE) was observed. The culture supernatant was collected by a centrifugation at 1,500 rpm and 4 °C for 5 min. The virus stock was kept as aliquots at −70 °C until use. Focus forming assay using PScloneD cells was performed to determine virus titer (FFU/ml) in the culture supernatant.

### DENV2 infection of endothelial cells

EA.hy926 cells were seeded in a 60-mm dish (Corning Costar; Cambridge, MA), on a coverslip, or on a collagen-coated polyethylene membrane insert (a pore size of 1.0 μm) in the Transwell^TM^ chamber (Millipore; Darmstadt, Germany) (type of the cell supporter/container was based on subsequent experiments) and were incubated until a monolayer was formed. The monolayer of EA.hy926 cells was infected with DENV2 at multiplicity of infection (MOI) of 2.0 and then incubated at 37 °C for 2 h in a humidified incubator with 5% CO_2_. After DENV2 challenge, the supernatant was removed and replaced with fresh maintenance DMEM/F-12 medium (containing 5% FBS). The cells were further incubated at 37 °C in a humidified incubator with 5% CO_2_ for the indicated time-points (12, 24, 36, 48 h post-infection) before analyses as follows. The non-infected cells maintained in parallel but without DENV2 challenge served as the mock-control.

### Western blot analysis

At 12, 24, 36, and 48 h post-infection, mock-control and DENV2-infected EA.hy926 cells in the 60-mm dishes were washed with ice-cold PBS five times. Cellular proteins were extracted using a lysis buffer containing 0.2 M Tris-HCl (pH 6.8), 20% β-mercaptoethanol, 8% sodium dodecyl sulfate (SDS), and 40% glycerol. Protein concentrations were quantitated by Bradford’s method using Bio-Rad Protein Assay (Bio-Rad; Milano, Italy). The plate well solution in lower chamber of Transwell was also collected at the same time-points. An equal amount of whole cell lysate (30 μg/sample) and equal volume of plate well solution in the lower chamber (3 μl/sample) were resolved by 12% SDS-PAGE and then transferred onto a nitrocellulose membrane (Whatman; Dassel, Germany) using a semidry transfer apparatus (Bio-Rad) at 75 mA for 90 min. Non-specific bindings were blocked with 10% (v/v) soy milk in PBS at room temperature (25 °C; RT) for 1 h. The membrane was then incubated with rabbit polyclonal anti-BSA (Santa Cruz Biotechnology; Santa Cruz, CA) or mouse monoclonal anti-GAPDH (Santa Cruz Biotechnology) (1:500 in 10% (v/v) soy milk/PBS) at 4 °C overnight. A mouse monoclonal antibody against DENV non-structural protein 1 (NS1) (Abcam; Cambridge, UK) was also used to detect NS1 as a marker for DENV infection. After incubation with primary antibody, the membrane was washed with PBS and further incubated at RT for 1 h with horseradish peroxidase-conjugated polyclonal swine anti-rabbit IgG or polyclonal rabbit anti-mouse IgG antibody (Dako; Denmark A/S, Denmark) (1:3,000 and 1:5,000, respectively, in 10% (v/v) soy milk/PBS). Reactive protein bands were visualized using SuperSignal West Pico chemiluminescence substrate (Pierce Biotechnology, Inc.; Rockford, IL).

### Detection of intracellular BSA conjugated with Alexa Fluor 488 (Alexa488-BSA) by laser scanning confocal microscopy

EA.hy926 cells were challenged with DENV2 on a coverslip as aforementioned. At 1 h prior to the harvest (12, 24, 36, 48 h post-infection), 300 μg of BSA conjugated with Alexa Fluor 488 (Alexa488-BSA) (Invitrogen; Grand Island, NY) was added into the medium and incubated with both mock and DENV2-infected cells for 1 h. At the indicated time-points, the cell monolayer was washed with ice-cold PBS five times, fixed with 3.7% formaldehyde in PBS for 10 min, and permeabilized with 1% Triton X-100 in PBS for 10 min at RT. The cells were then incubated with mouse monoclonal anti-DENV (9.F.10) (Santa Cruz Biotechnology) (1:50 in 1% BSA/PBS) at 4 °C overnight. Thereafter, donkey anti-mouse IgG conjugated with Alexa Fluor 555 (Invitrogen) (1:500 in 1% BSA/PBS) and Hoechst dye (Invitrogen) (1:500 in 1% BSA/ PBS) were added and further incubated with the cells at 37 °C for 1 h. The coverslip was mounted on a glass slide with 50% (v/v) glycerol in PBS and then visualized under a laser scanning confocal microscope (LSM 510 Meta, Carl Zeiss; Jena, Germany). The fluorescence intensity of intracellular Alexa Fluor 488-BSA was quantitatively analyzed from at least 10 random high-power fields (HPFs) using ImageJ software (version 1.48b) (http://imagej.nih.gov/ij/).

### Flow cytometric analysis of cell death

EA.hy926 cells were seeded on a collagen-coated polyethylene membrane insert (a pore size of 1.0 μm) in the Transwell^TM^ chamber and challenged with DENV2 at multiplicity of infection (MOI) of 2.0, 5.0 or 10.0 as aforementioned. At the indicated time-points (12, 24, 36, or 48 h post-infection), the cell monolayer was detached and collected by trypsinization with 0.1% trypsin and 2.5 mM EDTA in PBS. The cells were resuspended in the complete DMEM/F-12 medium (supplemented with 10% FBS) and pelleted by a centrifugation at 2,000 rpm for 5 min. After washing twice with ice-cold annexin V buffer (10 mM HEPES, 140 mM NaCl, 2.5 mM CaCl_2_.2H_2_O; pH 7.4), the cells were resuspended in annexin V buffer at a final concentration of 5 × 10^5^ cells/ml and then incubated with FITC-conjugated annexin V (BD Biosciences; San Diego, CA) on ice in the dark for 30 min. Propidium iodide (BD Biosciences) at a final concentration of 0.2 μg/ml was added to the cell suspension before analysis by flow cytometry using FACScan (BD Biosciences). The cells fixed with 3.7% formaldehyde in PBS for 10 min and permeabilized with 1% Triton X-100 in PBS for 10 min at RT were used as the positive control, while the untreated cells were used as the negative control.

### *In vitro* vascular permeability assay

This assay was performed using the Millipore *In Vitro* Vascular Permeability Assay Kit (ECM640) according to manufacturer’s instructions. Briefly, the cells were seeded on a collagen-coated polyethylene membrane insert (a pore size of 1.0 μm) in the Transwell^TM^ chamber and challenged with DENV2 as aforementioned. At the indicated time-points (12, 24, 36, 48 h post-infection), dextran conjugated with FITC (FITC-dextran) was added to the complete DMEM/F-12 medium (containing 10% FBS) and incubated at RT for 5 min. The insert was removed and plate well solution (lower chamber) was collected and measured for fluorescence intensity of FITC-dextran using Synergy HT microplate reader (Biotek Instruments, Inc.; Winooski, VT) with excitation and emission wavelengths of 485 and 530 nm, respectively.

### Quantitative analysis of permeated Alexa488-BSA

EA.hy926 cells were seeded on a collagen-coated polyethylene membrane insert (a pore size of 1.0 μm) in the Transwell^TM^ chamber and challenged with DENV2 as aforementioned, but with 10 μg Alexa488-BSA in each upper chamber, whereas the lower chamber was filled with 500 μl plain DMEM/F-12 medium (without FBS supplementation). At 12, 24, 36, and 48 h post-infection, the insert well solution (upper chamber) and plate well solution (lower chamber) were collected and measured for fluorescence intensity of Alexa488-BSA using Synergy HT microplate reader (Biotek Instruments) with excitation and emission wavelengths of 485 and 530 nm, respectively. The amount of permeated Alexa488-BSA was then calculated comparing to standard concentration curve. Fig. 3A summarizes the Transwell-based transcytosis assay.

### Inhibition of individual endocytic pathways

EA.hy926 cells were challenges with DENV2 on a coverslip as aforementioned. At 47 h post-infection, both mock and DENV2-infected cells were incubated for 10 min with each of various inhibitors, including 10 μM nystatin (Sigma), 10 μM chlorpromazine hydrochloride (Sigma), and 20 μM 5-(N-ethyl-N-isopropyl) amiloride (EIPA) (Sigma), to inhibit caveolae-dependent endocytosis, clathrin-coated vesicle-mediated endocytosis, and macropinocytosis, respectively. Thereafter, 300 μg of Alexa488-BSA (Invitrogen) was added into the medium and incubated with the cells for 50 min. At 48 h post-infection, the cells were washed with ice-cold PBS five times, fixed with 3.7% formaldehyde in PBS for 10 min, and permeabilized with 1% Triton X-100 in PBS at RT for 10 min. The cells were then incubated with mouse monoclonal anti-caveolin-1 (Santa Cruz Biotechnology) at 4 °C overnight. Thereafter, donkey anti-mouse-IgG conjugated with Alexa Fluor 555 (Invitrogen) (1:50 in 1% BSA/PBS) and Hoechst dye (Invitrogen) (1:500 in 1% BSA/PBS) were added and further incubated with the cells at 37 °C for 1 h. The coverslip was mounted on a glass slide with 50% (v/v) glycerol in PBS and then visualized under a laser scanning confocal microscope (LSM 510 Meta) with an excitation wavelength of 488 nm. The fluorescence intensity of intracellular Alexa488-BSA was quantitatively analyzed using Axiovision software (Carl Ziess).

### Statistical analysis

All quantitative data are presented as mean ± SD, unless stated otherwise. Comparison between two sample groups was performed using unpaired Student’s *t*-test, whereas multiple comparisons were performed using ANOVA with Tukey’s post-hoc test. P-values less than 0.05 were considered statistically significant.

## Additional Information

**How to cite this article**: Chanthick, C. *et al*. Caveolae-mediated albumin transcytosis is enhanced in dengue-infected human endothelial cells: A model of vascular leakage in dengue hemorrhagic fever. *Sci. Rep.* **6**, 31855; doi: 10.1038/srep31855 (2016).

## Supplementary Material

Supplementary Information
