# Supplementary material for: Caveolae-mediated albumin transcytosis is enhanced in dengue-infected human endothelial cells: A model of vascular leakage in dengue hemorrhagic fever
Source: Sci Rep. 2016 Aug 22;6:31855. doi: 10.1038/srep31855 (PMC4992822; doi:10.1038/srep31855)
Supplement: Supplementary Information [file srep31855-s1.pdf]

Caveolae-mediated albumin transcytosis is enhanced in dengue-infected human endothelial cells: A model of vascular leakage in dengue hemorrhagic fever

Chanettee Chanthick, Rattiyaporn Kanlaya, Rattanaporn Kiatbumrung, Sa-nga Pattanakitsakul, and Visith Thongboonkerd

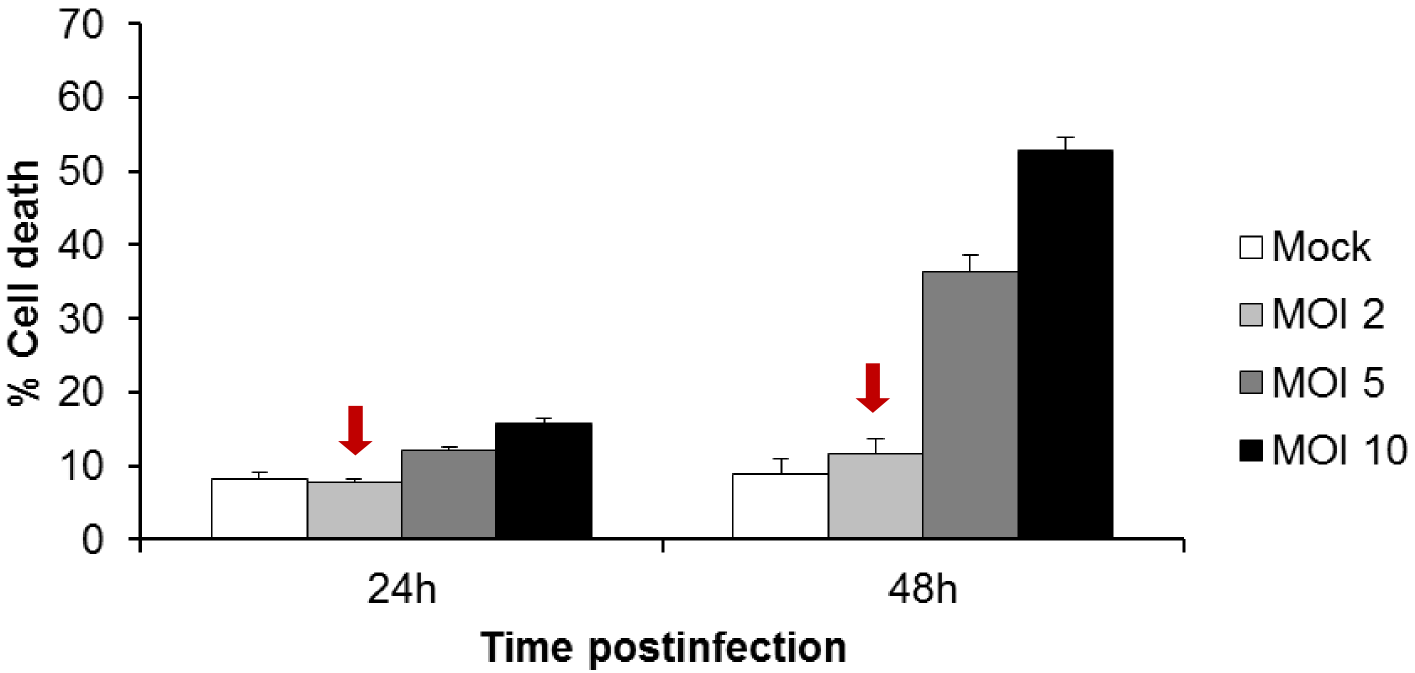

Supplementary Figure S1: Cell death was quantitated by flow cytometry using Annexin V/propidium iodide co-staining. Percentage of cell death was calculated using the formula: % cell death = [(no. of total cell death (apoptosis + necrosis)/no. of total cells) x 100%] (n = 3 independent experiments). Arrows indicate the optimal MOI (2.0) selected for all subsequent experiments.
